# Machine Learning and Natural Language Processing for Geolocation-Centric Monitoring and Characterization of Opioid-Related Social Media Chatter

**DOI:** 10.1001/jamanetworkopen.2019.14672

**Published:** 2019-11-06

**Authors:** Abeed Sarker, Graciela Gonzalez-Hernandez, Yucheng Ruan, Jeanmarie Perrone

**Affiliations:** 1 Department of Biostatistics, Epidemiology and Informatics, Perelman School of Medicine, University of Pennsylvania, Philadelphia; 2 Department of Biomedical Informatics, School of Medicine, Emory University, Atlanta, Georgia; 3 School of Engineering and Applied Science, University of Pennsylvania, Philadelphia; 4 Department of Emergency Medicine, Perelman School of Medicine, University of Pennsylvania, Philadelphia

## Abstract

**Importance:**

Automatic curation of consumer-generated, opioid-related social media big data may enable real-time monitoring of the opioid epidemic in the United States.

**Objective:**

To develop and validate an automatic text-processing pipeline for geospatial and temporal analysis of opioid-mentioning social media chatter.

**Design, Setting, and Participants:**

This cross-sectional, population-based study was conducted from December 1, 2017, to August 31, 2019, and used more than 3 years of publicly available social media posts on Twitter, dated from January 1, 2012, to October 31, 2015, that were geolocated in Pennsylvania. Opioid-mentioning tweets were extracted using prescription and illicit opioid names, including street names and misspellings. Social media posts (tweets) (n = 9006) were manually categorized into 4 classes, and training and evaluation of several machine learning algorithms were performed. Temporal and geospatial patterns were analyzed with the best-performing classifier on unlabeled data.

**Main Outcomes and Measures:**

Pearson and Spearman correlations of county- and substate-level abuse-indicating tweet rates with opioid overdose death rates from the Centers for Disease Control and Prevention WONDER database and with 4 metrics from the National Survey on Drug Use and Health for 3 years were calculated. Classifier performances were measured through microaveraged F1 scores (harmonic mean of precision and recall) or accuracies and 95% CIs.

**Results:**

A total of 9006 social media posts were annotated, of which 1748 (19.4%) were related to abuse, 2001 (22.2%) were related to information, 4830 (53.6%) were unrelated, and 427 (4.7%) were not in the English language. Yearly rates of abuse-indicating social media post showed statistically significant correlation with county-level opioid-related overdose death rates (n = 75) for 3 years (Pearson *r* = 0.451, *P* < .001; Spearman *r* = 0.331, *P* = .004). Abuse-indicating tweet rates showed consistent correlations with 4 NSDUH metrics (n = 13) associated with nonmedical prescription opioid use (Pearson *r* = 0.683, *P* = .01; Spearman *r* = 0.346, *P* = .25), illicit drug use (Pearson *r* = 0.850, *P* < .001; Spearman *r* = 0.341, *P* = .25), illicit drug dependence (Pearson *r* = 0.937, *P* < .001; Spearman *r* = 0.495, *P* = .09), and illicit drug dependence or abuse (Pearson *r* = 0.935, *P* < .001; Spearman *r* = 0.401, *P* = .17) over the same 3-year period, although the tests lacked power to demonstrate statistical significance. A classification approach involving an ensemble of classifiers produced the best performance in accuracy or microaveraged F1 score (0.726; 95% CI, 0.708-0.743).

**Conclusions and Relevance:**

The correlations obtained in this study suggest that a social media–based approach reliant on supervised machine learning may be suitable for geolocation-centric monitoring of the US opioid epidemic in near real time.

## Key Points

**Question:**

Can natural language processing be used to gain real-time temporal and geospatial information from social media data about opioid abuse?

**Findings:**

In this cross-sectional, population-based study of 9006 social media posts, supervised machine learning methods performed automatic 4-class classification of opioid-related social media chatter with a maximum F1 score of 0.726. Rates of automatically classified opioid abuse–indicating social media posts from Pennsylvania correlated with county-level overdose death rates and with 4 national survey metrics at the substate level.

**Meaning:**

The findings suggest that automatic processing of social media data, combined with geospatial and temporal information, may provide close to real-time insights into the status and trajectory of the opioid epidemic.

## Teaser

This cross-sectional study develops and validates a machine learning method for collecting and classifying data from opioid-related postings on a social media platform.

## Introduction

The problem of drug addiction and overdose has reached epidemic proportions in the United States, and it is largely driven by opioids, both prescription and illicit.^1^ More than 72 000 overdose-related deaths in the United States were estimated to have occurred in 2017, of which more than 47 000 (approximately 68%) involved opioids,^2^ meaning that a mean of more than 130 people died each day from opioid overdoses, and approximately 46 of these deaths were associated with prescription opioids.^3^ According to the Centers for Disease Control and Prevention, the opioid crisis has hit some US states harder than others, with West Virginia, Ohio, and Pennsylvania having death rates greater than 40 per 100 000 people in 2017 and with statistically significant increases in death rates year by year.^4^ Studies have suggested that the state-by-state variations in opioid overdose–related deaths are multifactorial but may be associated with differences in state-level policies and laws regarding opioid prescribing practices and population-level awareness or education regarding the risks and benefits of opioid use.^5^ Although the geographic variation is now known, strategies for monitoring the crisis are grossly inadequate.^6,7^ Current monitoring strategies have a substantial time lag, meaning that the outcomes of recent policy changes, efforts, and implementations^8,9,10^ cannot be assessed close to real time. Kolodny and Frieden^11^ discussed some of the drawbacks of current monitoring strategies and suggested 10 federal-level steps for reversing the opioid epidemic, with improved monitoring or surveillance as a top priority.

In recent years, social media has emerged as a valuable resource for performing public health surveillance,^12,13,14,15^ including for drug abuse.^16,17,18^ Adoption of social media is at an all-time high^19^ and continues to grow. Consequently, social media chatter is rich in health-related information, which, if mined appropriately, may provide unprecedented insights. Studies have suggested that social media posts mentioning opioids and other abuse-prone substances contain detectable signals of abuse or misuse,^20,21,22^ with some users openly sharing such information, which they may not share with their physicians or through any other means.^13,17,23,24^ Manual analyses established the potential of social media for drug abuse research, but automated, data-centric processing pipelines are required to fully realize social media’s research potential. However, the characteristics of social media data present numerous challenges to automatic processing from the perspective of natural language processing and machine learning, including the presence of misspellings, colloquial expressions, data imbalance, and noise. Some studies have automated social media mining for this task by proposing approaches such as rule-based categorization,^22^ supervised classification,^17^ and unsupervised methods.^5^ Studies that have compared opioid-related chatter and its association with the opioid crisis have been unsupervised in nature, and they either do not filter out information unrelated to personal abuses^5^ or do not quantitatively evaluate the performance of their filtering strategy.^21^ These and similar studies have, however, established the importance of social media data for toxicovigilance and have paved the platform for end-to-end automatic pipelines for using social media information in near real time.

In this cross-sectional study, we developed and evaluated the building blocks, based on natural language processing and machine learning, for an automated social media–based pipeline for toxicovigilance. The proposed approach relies on supervised machine learning to automatically characterize opioid-related chatter and combines the output of the data processing pipeline with temporal and geospatial information from Twitter to analyze the opioid crisis at a specific time and place. We believe this supervised learning-based model is more robust than unsupervised approaches as it is not dependent on the volume of the overall chatter, which fluctuates from time to time depending on various factors, such as media coverage. This study, which focused on the state of Pennsylvania, suggests that the rate of personal opioid abuse–related chatter on Twitter was reflective of the opioid overdose deaths from the Centers for Disease Control and Prevention WONDER database and 4 metrics from the National Surveys on Drug Use and Health (NSDUH) over a period of 3 years.

## Methods

### Data Collection, Refinement, and Annotation

This cross-sectional study was conducted from December 1, 2017, to August 31, 2019. It was deemed by the University of Pennsylvania Institutional Review Board to be exempt from review as all data used were publicly available. Informed consent was not necessary for this reason. This study followed the Strengthening the Reporting of Observational Studies in Epidemiology (STROBE) reporting guideline.

Publicly available social media posts on Twitter from January 1, 2012, to October 31, 2015, were collected as part of a broader project through the public streaming API (application programming interface).^25^ The API provides access to a representative random sample of approximately 1% of the data in near real time.^26^ Social media posts (tweets) originating from Pennsylvania were identified through the geolocation detection process, as described in Schwartz et al.^27^ To include opioid-related posts only, our research team, led by a medical toxicologist (J.P.), identified keywords, including street names (relevant unambiguous street names were chosen from the US Drug Enforcement Administration website^28^) that represented prescription and illicit opioids. Because social media posts have been reported to include many misspellings,^29^ and drug names are often misspelled, we used an automatic spelling variant generator for the selected keywords.^30^ We observed an increase in retrieval rate for certain keywords when we combined these misspellings with the original keywords (example in eFigure 1 in the Supplement).

We wanted to exclude noisy terms with low signal to noise ratios for the manual annotation phase. We manually analyzed a random sample of approximately 16 000 social media posts to identify such noisy terms. We found that 4 keywords (*dope*, *tar*, *skunk*, and *smack*) and their spelling variants occurred in more than 80% of the tweets (eFigure 2 in the Supplement). Manual review performed by one of us (A.S.) and the annotators suggested that almost all social media posts retrieved by these keywords were referring to nonopioid content. For example, the term *dope* is typically used in social media to indicate something is good (eg, “that song is dope”). We removed all the posts mentioning these keywords, which reduced the data set from more than 350 000 to approximately 131 000, a decrease of more than 50%.

We developed annotation guidelines using the grounded theory approach.^31^ First, we grouped tweets into topics and then into broad categories. Four annotation categories or classes were chosen: self-reported abuse or misuse (A), information sharing (I), unrelated (U), and non-English (E). Iterative annotation of a smaller set of 550 posts was used to develop the guidelines and to increase agreement between the annotators. For the final annotation set, disagreements were resolved by a third annotator. Further details about the annotation can be found in the pilot publication^32^ and eTable 1 in the Supplement.

### Machine Learning Models and Classification

We used the annotated posts to train and evaluate several supervised learning algorithms and to compare their performances. We experimented with 6 classifiers: naive bayes, decision tree, k-nearest neighbors, random forest, support vector machine, and a deep convolutional neural network. Tweets were preprocessed before training or evaluation by lowercasing. For the first 5 of the 6 classifiers (or traditional classifiers), we stemmed the terms as a preprocessing step using the Porter stemmer.^33^ As features for the traditional classifiers, we used word n-grams (contiguous sequences of words) along with 2 additional engineered features (word clusters and presence and counts of abuse-indicating terms) that we had found to be useful in our related past work.^17^ The sixth classifier, a deep convolutional neural network, consisted of 3 layers and used dense vector representations of words, commonly known as *word embeddings*,^34^ which were learned from a large social media data set.^35^ Because the word embeddings we used were learned from social media drug-related chatter, they captured the semantic representations of drug-related keywords.

We randomly split the annotated posts into 3 sets: training, validation, and testing. For parameter optimization of the traditional classifiers, we combined the training and validation sets and identified optimal parameter values by using 10-fold cross-validations (eTable 2 in the Supplement). For the deep convolutional neural network, we used the validation set at training time for finding optimal parameter values, given that running 10-fold cross-validation for parameter optimization of neural networks is time consuming and hence infeasible. The best performance achieved by each classifier over the training set is presented in eTable 3 in the Supplement. To address the data imbalance between classes, we evaluated each individual classifier using random undersampling of the majority class (U) and oversampling of the pertinent smaller classes (A and I) using SMOTE (synthetic minority oversampling technique^36^).

In addition, we used ensembling strategies for combining the classifications of the classifiers. The first ensembling method was based on majority voting; the most frequent classification label by a subset of the classifiers was chosen as the final classification. In the case of ties, the classification by the best-performing individual classifier was used. For the second ensembling approach, we attempted to improve recall for the 2 nonmajority classes (A and I), which represented content-rich posts. For this system variant, if any post was classified as A or I by at least 2 classifiers, the post was labeled as such. Otherwise, the majority rule was applied.

We used the best-performing classification strategy for all the unlabeled posts in the data set. Our goal was to study the distributions of abuse- and information-related social media chatter over time and geolocations, as past research has suggested that such analyses may reveal interesting trends.^5,21,37^

### Statistical Analysis

We compared the performances of the classifiers using the precision, recall, and microaveraged F1 or accuracy scores. The formulas for computing the metrics were as follows, with tp representing true positives; fn, false negatives; and fp, false-positives:


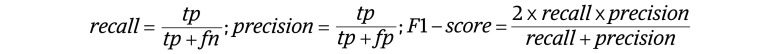
.

To compute the microaveraged F1 score, the tp, fp, and fn values for all of the classes are summed before calculating precision and recall. Formally, 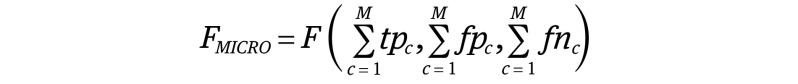,in which *F* is the function to compute the metric, *c* is a label, and *M* is the set of all labels. For a multiclass problem such as this, microaveraged F1 score and accuracy are equal. We computed 95% CIs for the F1 scores using the bootstrap resampling technique^38^ with 1000 resamples.

For geospatial analyses, we compared the abuse-indicating social media post rates from Pennsylvania with related metrics for the same period from 2 reference data sets: the WONDER database^39^ and the NSDUH.^40^ We obtained county-level yearly opioid overdose death rates from WONDER and percentages for 4 relevant substate-level measures (past month use of illicit drugs [no marijuana], past year nonmedical use of pain relievers, past year illicit drug dependence or abuse, and past year illicit drug dependence) from NSDUH. All the data collected were for the years 2012 to 2015. For the NSDUH measures, percentage values of annual means over the 3 years were obtained. We investigated the possible correlations (Pearson and Spearman) between the known metrics and the automatically detected abuse-indicating tweet rates and then visually compared them using geospatial heat maps and scatterplots.

For Pearson and Spearman correlation analyses, we used the Python library SciPy, version 1.3.1. Two-tailed *P* < .05 was interpreted as statistical significance.

## Results

We used 56 expressions of illicit and prescription opioids for data collection, with a total of 213 keywords or phrases, including spelling variants (eTable 4 in the Supplement). The annotations resulted in a final set of 9006 social media posts (6304 [70.0%] for training, 900 [10.0%] for validation, and 1802 [20.0%] for testing). There were 550 overlapping posts between the 2 annotators, and interannotator agreement was moderate with κ = 0.75 (Cohen κ^41^). Of the 9006 posts, 4830 (53.6%) were unrelated to opioids, 427 (4.7%) were not in the English language, and the proportions of abuse (1748 [19.4%]) and information (2001 [22.2%]) posts were similar (eTable 5 in the Supplement).

To capture the natural variation in the distribution of posts in real time, we did not stratify the sets by class during the training or testing set splitting. Consequently, the testing set consisted of a marginally lower proportion of abuse-indicating posts (17.7%) compared with the training set (19.8%). Statistically significant variation was found in the distribution of posts mentioning prescriptions (2257 [25.1%]) and illicit opioids (7038 [78.1%]) at an approximate ratio of 3:1. Proportions of class A and class I tweets were much higher for prescription opioid tweets (24.7% vs 18.0% for class A; 30.4% vs 20.9% for class I), whereas the proportion of class U tweets (55.1% vs 44.5%) was much higher for the illicit opioid posts (see eTable 5 in the Supplement for post distributions per class).

### Model Performances

Table 1 presents the performances of the classification algorithms, showing the recall, precision, and microaveraged F1 score and 95% CIs. Among the traditional classifiers, support vector machines (F1 score = 0.700; 95% CI, 0.681-0.718) and random forests (F1 score = 0.701; 95% CI, 0.683-0.718) showed similar performances, outperforming the others in F1 scores. The deep convolutional neural network outperformed all of the traditional classifiers (F1 score = 0.720; 95% CI, 0.699-0.735). The resampling experiments did not improve performance of the individual classifiers. Both pairs of ensemble classification strategies shown in Table 1 performed better than the individual classifiers, with the simple majority voting ensemble of 4 classifiers (Ensemble_1) producing the best microaveraged F1 score (0.726; 95% CI, 0.708-0.743). Performances of the classifiers were high for class U and class N and low for class A.

**Table 1.** Performances of Different Classifiers on the Testing Set

Classifier	Precision				Recall				Microaveraged F1 or Accuracy Score (95% CI)
	Class A	Class I	Class U	Class N	Class A	Class I	Class U	Class N	
Random classifier^a^	0.166	0.235	0.535	0.052	0.189	0.224	0.530	0.044	0.375 (0.360-0.394)
NB	0.307	0.501	0.788	0.737	0.670	0.504	0.463	0.811	0.539 (0.518-0.558)
NB Random oversampling	0.297	0.502	0.806	0.745	0.695	0.495	0.456	0.778	0.523 (0.505-0.542)
NB Undersampling	0.293	0.620	0.820	0.735	0.733	0.454	0.499	0.867	0.548 (0.529-0.568)
NB SMOTE	0.319	0.509	0.793	0.737	0.651	0.498	0.526	0.811	0.555 (0.536-0.574)
DT	0.389	0.540	0.725	0.816	0.371	0.447	0.783	0.889	0.638 (0.618-0.655)
DT Random oversampling	0.388	0.510	0.752	0.818	0.455	0.476	0.724	0.900	0.617 (0.599-0.644)
DT Undersampling	0.341	0.481	0.797	0.802	0.487	0.548	0.630	0.900	0.599 (0.579-0.617)
DT SMOTE	0.307	0.437	0.723	0.833	0.365	0.488	0.638	0.889	0.568 (0.549-0.587)
k-NN	0.314	0.791	0.589	0.852	0.101	0.081	0.942	0.876	0.593 (0.574-0.612)
k-NN Random oversampling	0.287	0.629	0.627	0.861	0.248	0.159	0.852	0.900	0.587 (0.567-0.607)
k-NN Undersampling	0.355	0.474	0.815	0.781	0.522	0.572	0.606	0.911	0.599 (0.580-0.618)
k-NN SMOTE	0.317	0.446	0.724	0.868	0.380	0.493	0.643	0.878	0.574 (0.549-0.587)
SVM	0.476	0.717	0.728	0.895	0.374	0.529	0.856	0.944	0.700 (0.681-0.718)
SVM Random oversampling	0.446	0.657	0.821	0.895	0.560	0.756	0.644	0.944	0.704 (0.683 –0.720)
SVM Undersampling	0.409	0.611	0.862	0.843	0.629	0.668	0.667	0.956	0.675 (0.656 0.693)
SVM Oversampling SMOTE	0.330	0.598	0.764	0.920	0.566	0.548	0.616	0.9	0.605 (0.587-0.624)
RF	0.493	0.762	0.713	0.835	0.330	0.469	0.897	0.956	0.701 (0.683-0.718)
RF Random oversampling	0.447	0.679	0.775	0.835	0.462	0.569	0.809	0.956	0.700 (0.684-0.719)
RF Undersampling	0.414	0.561	0.883	0.791	0.616	0.688	0.639	0.967	0.663 (0.645-0.682)
RF Oversampling SMOTE	0.379	0.539	0.771	0.843	0.465	0.565	0.688	0.956	0.634 (0.616-0. 652)
CNN	0.532	0.676	0.759	0.902	0.386	0.608	0.858	0.922	0.720 (0.699-0.735)
CNN Random oversampling	0.532	0.677	0.758	0.902	0.386	0.602	0.860	0.922	0.720 (0.699-0.734)
CNN Undersampling	0.414	0.551	0.866	0.902	0.400	0.565	0.639	0.922	0.638 (0.618-0.658)
CNN SMOTE	0.493	0.598	0.800	0.902	0.414	0.548	0.688	0.922	0.658 (0.640-0.677)
Ensemble_1 (CNN, RF, SVM, NB)	0.517	0.721	0.758	0.887	0.425	0.565	0.866	0.956	0.726 (0.708-0.743)^b^
Ensemble_biased_1 (CNN, RF, SVM, NB)	0.489	0.716	0.780	0.887	0.506	0.563	0.836	0.956	0.721 (0.703-0.739)
Ensemble_2 (CNN, RF, SVM, NB, DT)	0.482	0.707	0.743	0.878	0.377	0.517	0.875	0.956	0.709 (0.692-0.726)
Ensemble_biased_2 (CNN, RF, SVM, NB, DT)	0.456	0.708	0.810	0.878	0.597	0.577	0.786	0.956	0.713 (0.696-0.730)

Abbreviations: A, self-reported abuse or misuse; CNN, convolutional neural network; DT, decision tree; I, information sharing; k-NN, k-nearest neighbors; N, non-English; NB, naive Bayes; RF, random forest; SMOTE, synthetic minority oversampling technique; SVM, support vector machine; U, unrelated.

^a^ The random classifier randomly assigns 1 of the 4 classes to a tweet.

^b^ Best performance.

The most common errors for the best-performing system (Ensemble_1) were incorrect classification to class U, comprising 145 (79.2%) of the 183 incorrect classifications for posts originally labeled as class A, 122 (67.4%) of the 181 incorrect classifications for posts labeled as class I, and all 4 (100%) of the incorrect classifications for posts labeled as class N (eTable 7 in the Supplement).

### Temporal and Geospatial Analyses

**Figure 1.**
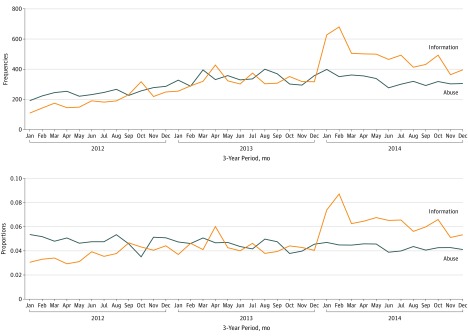
Monthly Distributions of the Frequencies and Proportions of Social Media Posts Classified as Abuse and Information in the Unlabeled Data Set Over 3 Years

Figure 1 shows the monthly frequency and proportion distributions of class A and I posts. The frequencies of both categories of posts increased over time, which was unsurprising given the growth in the number of daily active Twitter users over the 3 years of study as well as greater awareness about the opioid crisis. Greater awareness is perhaps also reflected by the increasing trend in information-related tweets. However, although the volume of abuse-related chatter increased, its overall proportion in all opioid-related chatter decreased over time, from approximately 0.055 to approximately 0.042. The true signals of opioid abuse from social media were likely hidden in large volumes of other types of information as awareness about the opioid crisis increased.

**Figure 2.**
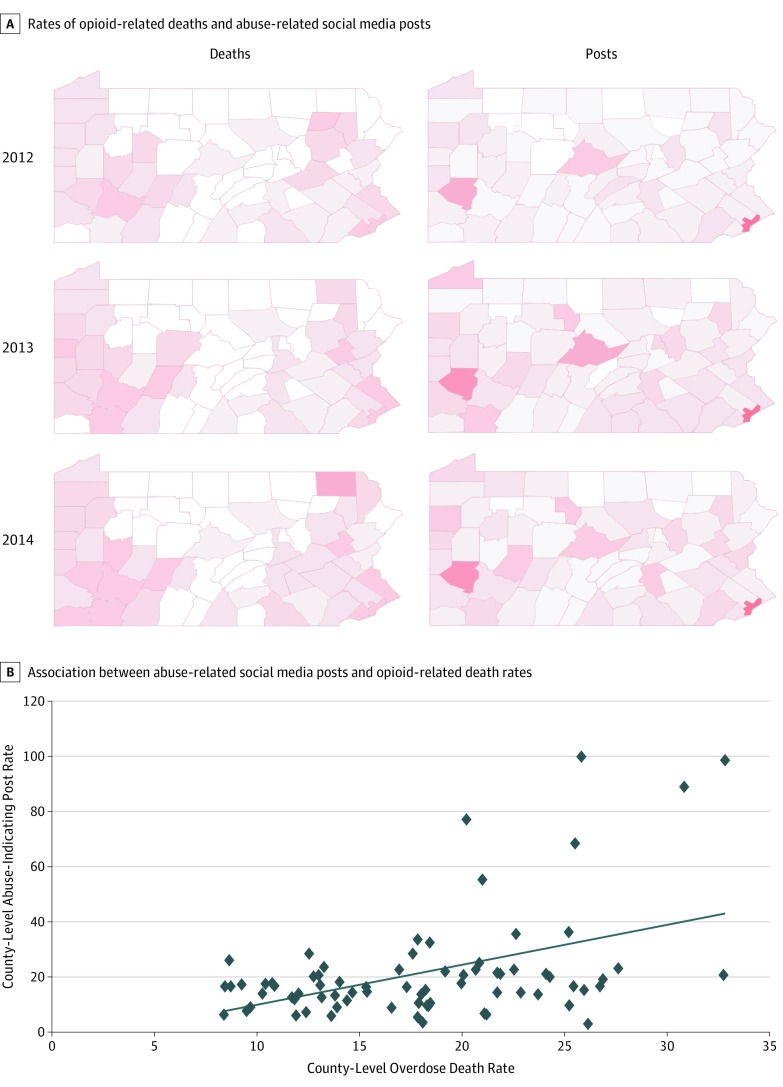
Comparison of County-Level Heat Maps of Opioid-Related Death Rates and Abuse-Related Social Media Post Rates in Pennsylvania, 2012-2014, and Scatterplot of the Association Between the 2 Variables

Figure 2 shows the similarities between 2 sets of county-level heat maps for population-adjusted, overdose-related death rates and abuse-indicating post rates as well as a scatterplot illustrating the positive association between the 2 variables. We found a statistically significant correlation (Pearson *r* = 0.451, *P* < .001; Spearman *r* = 0.331, *P* = .004) between the county-level overdose death rates and the abuse-indicating social media posts over 3 years (n = 75). In comparison, the pioneering study by Graves et al,^5^ perhaps the study most similar to ours, reported a maximum (among 50 topics) Pearson correlation of 0.331 between a specific opioid-related social media topic and county-level overdose death rates. In addition, we found that the Pearson correlation coefficient increased when the threshold for the minimum number of deaths for including counties was raised. If only counties with at least 50 deaths were included, the Pearson correlation coefficient increased to 0.54; for 100 deaths, the correlation coefficient increased to 0.67.

**Figure 3.**
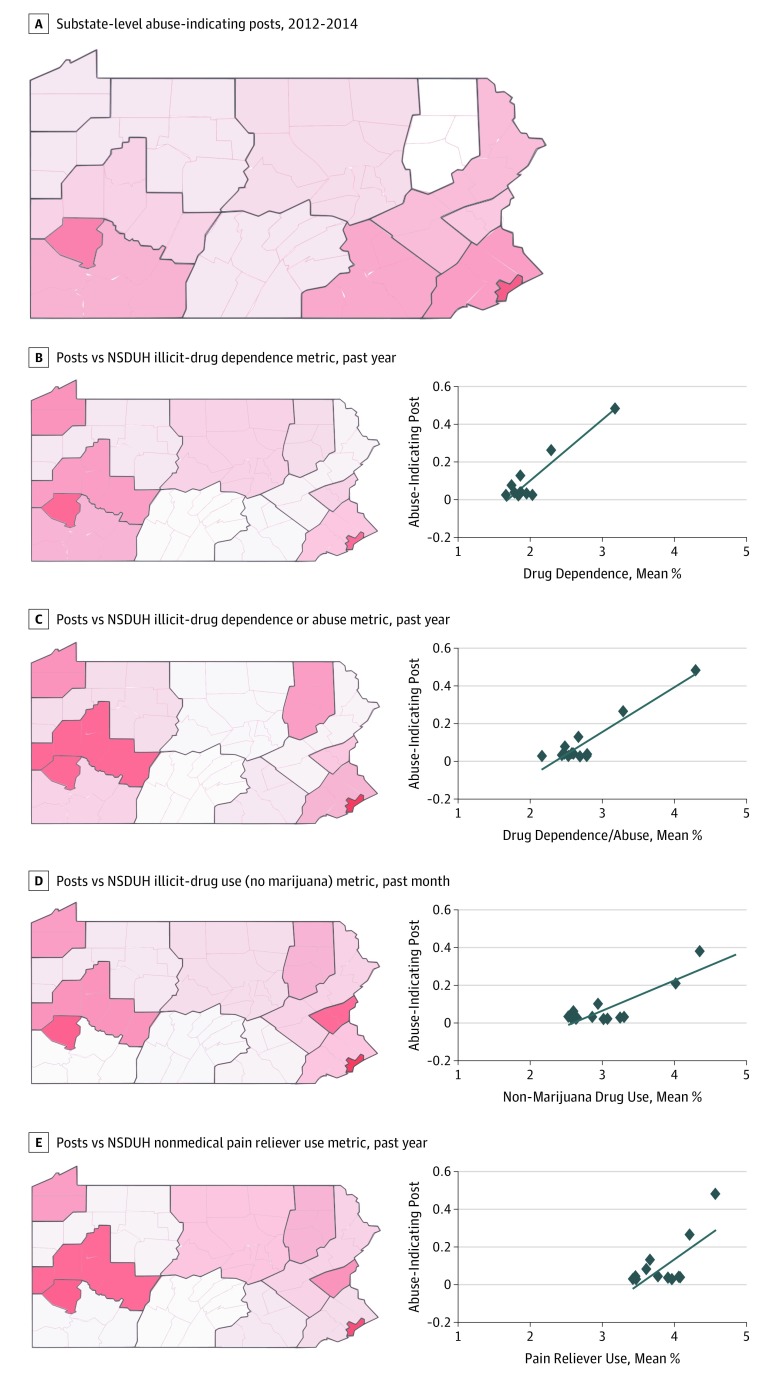
Substate-Level Heat Maps and Scatterplots Comparing Frequencies of Abuse-Indicating Social Media Posts With 4 Survey Metrics, 2012-2014 The computed correlations and their statistical significance are summarized in Table 2. Pennsylvania substate information is found in eTable 6 in the Supplement. NSDUH indicates National Survey on Drug Use and Health.

Figure 3 shows the substate-level heat maps for abuse-indicating social media posts and 4 NSDUH metrics over the same 3-year period, along with scatterplots for the 2 sets of variables. All the computed correlations and their significances are summarized in Table 2 (see eTable 6 in the Supplement for the substate information). Table 2 illustrates the consistently high correlations between abuse-indicating social media post rates and the NSDUH survey metrics over the same 3-year period (n = 13): nonmedical prescription opioid use (Pearson *r* = 0.683, *P* = .01; Spearman *r* = 0.346, *P* = .25), illicit drug use (Pearson *r* = 0.850, *P* < .001; Spearman *r* = 0.341, *P* = .25), illicit drug dependence (Pearson *r* = 0.937, *P* < .001; Spearman *r* = 0.495, *P* = .09), and illicit drug dependence or abuse (Pearson *r* = 0.935, *P* < .001; Spearman *r* = 0.401, *P* = .17). However, we could not establish statistical significance owing to the small sample sizes.

**Table 2.** Pearson and Spearman Correlations for Geolocation-Specific Abuse-Indicating Social Media Post Rates With County-Level Opioid Overdose Death Rates and 4 Metrics From the National Survey on Drug Use and Health

Measure	Pearson *r*	*P* Value	Spearman *r*	*P* Value	No. of Data Points
Opioid overdose death rate	0.451	<.001^a^	0.331	.004^a^	75
Illicit drug use, no marijuana, past mo	0.850	<.001^a^	0.341	.25	13
Nonmedical use of pain relievers, past y	0.683	.01	0.346	.25	13
Illicit drug dependence or abuse, past y	0.935	<.001^a^	0.401	.17	13
Illicit drug dependence, past y	0.937	<.001^a^	0.495	.09	13

^a^ Indicates statistical significance.

## Discussion

Opioid misuse or abuse and addiction are among the most consequential and preventable public health threats in the United States.^42^ Social media big data, coupled with advances in data science, present a unique opportunity to monitor the problem in near real time.^20,37,43,44,45^ Because of varying volumes of noise in generic social media data, the first requirement we believe needs to be satisfied for opioid toxicosurveillance is the development of intelligent, data-centric systems that can automatically collect and curate data, a requirement this cross-sectional study addressed. We explored keyword-based data collection approaches and proposed, through empirical evaluations, supervised machine learning methods for automatic categorization of social media chatter on Twitter. The best F1 score achieved was 0.726, which was comparable to human agreement.

Recent studies have investigated potential correlations between social media data and other sources, such as overdose death rates^5^ and NSDUH survey metrics.^21^ The primary differences between the current work and past studies are that we used a more comprehensive data collection strategy by incorporating spelling variants, and we applied supervised machine learning as a preprocessing step. Unlike purely keyword-based or unsupervised models,^5,46,47^ the approach we used appears to be robust at handling varying volumes of social media chatter, which is important when using social media data for monitoring and forecasting, given that the volume of data can be associated with factors such as movies or news articles, as suggested by Figure 1. The heat maps in Figures 2 and 3 show that the rates of abuse-related chatter were much higher in the more populous Pennsylvania counties (eg, Philadelphia and Allegheny), which was likely related to the social media user base being skewed to large cities. More advanced methods for adjusting or normalizing the data in large cities may further improve the correlations.

We also found that the correlation coefficient tended to increase when only counties with higher death rates were included. This finding suggests that Twitter-based classification may be more reliable for counties or geolocations with higher populations and therefore higher numbers of users. If this assertion is true, the increasing adoption of social media in recent years, specifically Twitter, is likely to aid the proposed approach. The correlations between social media post rates and the NSDUH metrics were consistently high, but statistical significance could not be established owing to the smaller sample sizes.

The proposed model we present in this study enables the automatic curation of opioid misuse–related chatter from social media despite fluctuating numbers of posts over time. The outputs of the proposed approach correlate with related measures from other sources and therefore may be used for obtaining near-real-time insights into the opioid crisis or for performing other analyses associated with opioid misuse or abuse.

### Classification Error Analysis

As mentioned, the most common error made by the best-performing classifier (Ensemble_1) was to misclassify social media posts to class U, whereas misclassifications to the other 3 classes occurred with much lower frequencies (eTable 7 in the Supplement). We reviewed the confusion matrices from the other classifiers and saw a similar trend. Because class U was the majority class, by a margin, it was the category to which the classifiers tended to group posts that lacked sufficient context. Short lengths of certain posts and the presence of misspellings or rare nonstandard expressions added difficulty for the classifiers to decipher contextual cues, a major cause of classification errors.

Lack of context in posts also hindered the manual annotations, making the categorizations dependent on the subjective assessments of the annotators. Although the final agreement level between the annotators was higher than the levels in initial iterations, it could be improved. Our previous work suggests that preparing thorough annotation guidelines and elaborate annotation strategies for social media–based studies helps in obtaining relatively high annotator agreement levels and, eventually, improved system performances.^48,49^ We plan to address this issue in future research.

Another factor that affected the performance of the classifiers on class A and class I was data imbalance; the relatively low number of annotated instances for these classes made it difficult for algorithms to optimally learn. The resampling experiments were not associated with improved performances, which is consistent with findings from past research.^49,50^ Annotating more data is likely to produce improved performances for these classes. Given that several recent studies obtained knowledge from Twitter about opioid use or abuse, combining all the available data in a distant supervision framework may be valuable.^51^ We will also explore the use of sentence-level contextual embeddings, which have been shown to outperform past text classification approaches.^52^

In future research, we plan to expand this work to other classes of drugs and prescription medications, such as stimulants and benzodiazepines. Combining machine learning and available metadata, we will estimate the patterns of drug consumption and abuse over time and across geolocations and analyze cohort-level data, building on our previous work.^53^

### Limitations

This cross-sectional study has several limitations. First, we included social media posts that originated from Pennsylvania. The advantage of machine learning over rule-based approaches is portability, but the possibly differing contents of social media chatter in different geolocations may reduce machine learning performance unless additional training data are added. Social media chatter is also always evolving, with new expressions introduced constantly. Therefore, systems trained with data from specific periods and geolocations may not perform optimally for other periods. The use of dense vector-based representations of texts may address this problem as semantic representations of emerging terms may be learned from large, unlabeled data sets without requiring human annotations.

Second, the moderate interannotator agreement in this study provided a relatively low ceiling for the machine learning classifier performance. More detailed annotation guidelines and strategies may address this problem by making the annotation process less subjective. Furthermore, the correlations we obtained did not necessarily indicate any higher-level associations between abuse-related social media posts and overdose death rates and/or survey responses.

## Conclusions

Big data derived from social media such as Twitter present the opportunity to perform localized monitoring of the opioid crisis in near real time. In this cross-sectional study, we presented the building blocks for such social media–based monitoring by proposing data collection and classification strategies that employ natural language processing and machine learning.

## Notes

Supplement. **eFigure 1.** Frequencies of Misspellings of Six Opioid Keywords Relative to the Frequencies of the Original Spellings **eFigure 2.** Distribution of Opioid-Related Keywords in a Sample of 16,320 Tweets **eTable 1.** Definitions of the Four Annotation Categories **eTable 2.** Optimal Parameter Values for the Different Classifiers Presented **eTable 3.** Class-Specific Recall and Precision, Average Accuracy and Standard Deviation Over Ten Folds for Each Classifier **eTable 4.** Opioid Keywords and Spelling Variants **eTable 5.** Distribution of Tweet Classes Across the Training and the Evaluation Sets **eTable 6.** Counties Within Each Substate in Pennsylvania **eTable 7.** Confusion Matrices Illustrating Common Errors Made by the 2 Best Performing Systems (Ensemble_1 and Ensemble_biased_1 in Table 1)
