# Supplementary material for: Machine Learning and Natural Language Processing for Geolocation-Centric Monitoring and Characterization of Opioid-Related Social Media Chatter
Source: JAMA Netw Open. 2019 Nov 6;2(11):e1914672. doi: 10.1001/jamanetworkopen.2019.14672 (PMC6865282; doi:10.1001/jamanetworkopen.2019.14672)
Supplement: Supplement. — eFigure 1. Frequencies of Misspellings of Six Opioid Keywords Relative to the Frequencies of the Original Spellings eFigure 2. Distribution of Opioid-Related Keywords in a Sample of 16,320 Tweets eTable 1. Definitions of the Four Annotation Categories eTable 2. Optimal Parameter Values for the Different Classifiers Presented eTable 3. Class-Specific Recall and Precision, Average Accuracy and Standard Deviation Over Ten Folds for Each Classifier eTable 4. Opioid Keywords and Spelling Variants eTable 5. Distribution of Tweet Classes Across the Training and the Evaluation Sets eTable 6. Counties Within Each Substate in Pennsylvania eTable 7. Confusion Matrices Illustrating Common Errors Made by the 2 Best Performing Systems (Ensemble_1 and Ensemble_biased_1 in Table 1) [file jamanetwopen-2-e1914672-s001.pdf]

Supplementary Online Content

Sarker A, Gonzalez-Hernandez G, Ruan Y, Perrone J. Machine learning and natural language processing for geolocation-centric monitoring and characterization of opioid-related social media chatter. *JAMA Netw Open.* 2019;2(11):e1914672. doi:10.1001/jamanetworkopen.2019.14672

eFigure 1. Frequencies of Misspellings of Six Opioid Keywords Relative to the Frequencies of the Original Spellings

eFigure 2. Distribution of Opioid-Related Keywords in a Sample of 16,320 Tweets

eTable 1. Definitions of the Four Annotation Categories

eTable 2. Optimal Parameter Values for the Different Classifiers Presented

eTable 3. Class-Specific Recall and Precision, Average Accuracy and Standard Deviation Over Ten Folds for Each Classifier

eTable 4. Opioid Keywords and Spelling Variants

eTable 5. Distribution of Tweet Classes Across the Training and the Evaluation Sets

eTable 6. Counties Within Each Substate in Pennsylvania

eTable 7. Confusion Matrices Illustrating Common Errors Made by the 2 Best Performing Systems (Ensemble_1 and Ensemble_biased_1 in Table 1)

This supplementary material has been provided by the authors to give readers additional information about their work.

eFigure 1. Frequencies of Misspellings of Six Opioid Keywords Relative to the Frequencies of the Original Spellings

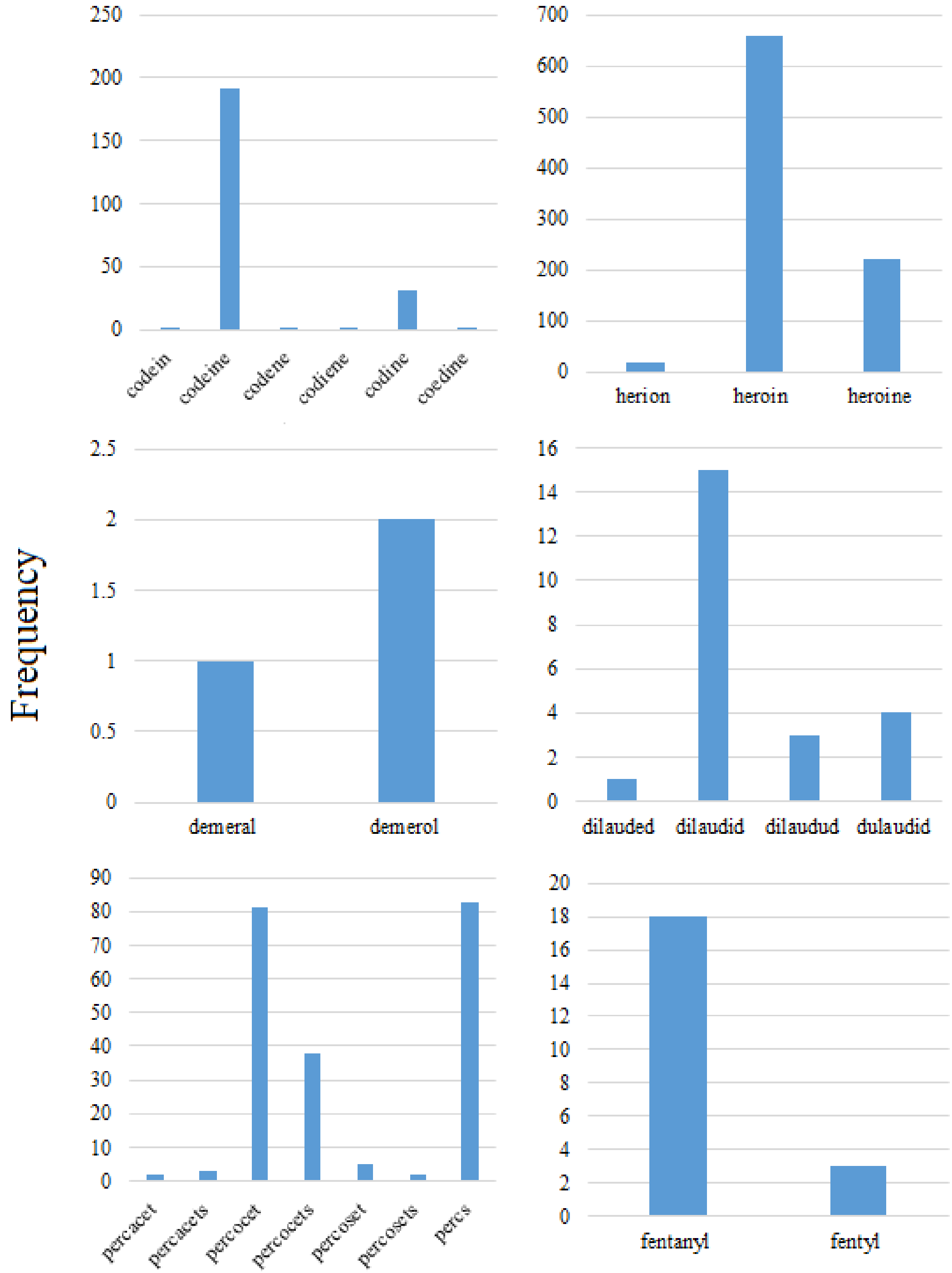

© 2019 Sarker A et al. *JAMA Network Open*

eFigure 2. Distribution of Opioid-Related Keywords in a Sample of 16,320 Tweets

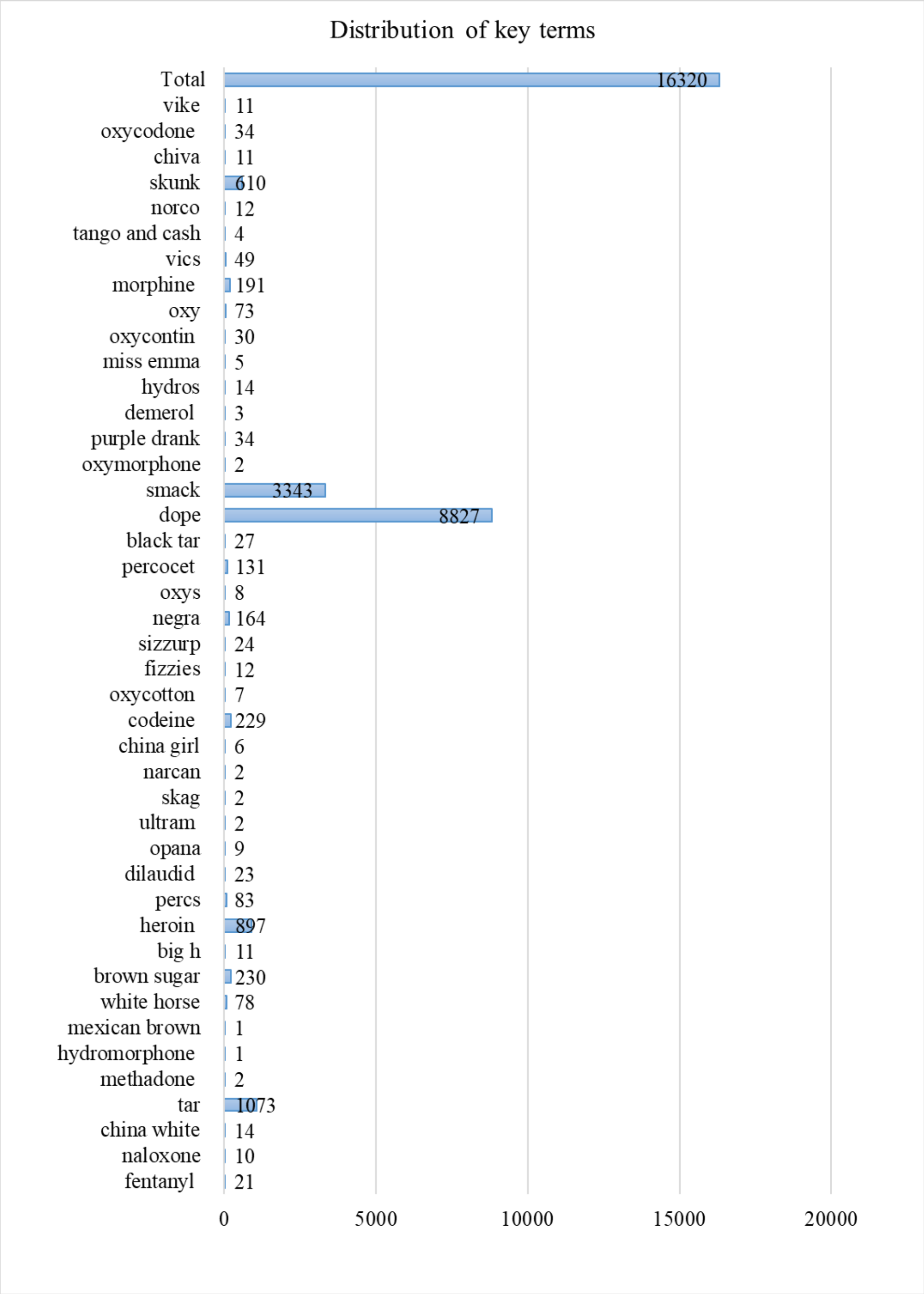

© 2019 Sarker A et al. *JAMA Network Open*

eTable 1. Definitions of the Four Annotation Categories

Note: User handles have been replaced with the generic tag @USERNAME to protect the identity of the users. Offensive words have been replaced by '*', URLs have been replaced with the tag [URL].

<table>
<tr><th>Category</th><th>Definition</th></tr>
<tr><td>Abuse/misuse-related (A)</td><td>This category includes abuse-indicating or possible abuse by the poster of the tweet or by someone the poster knows or is communicating with. Possible abuse are posts that do not explicitly state opioid abuse, but the tweets contain hints of current or past abuse.

Admissions of abuse in the past and expressions suggesting the illicit online trading of opioids are also included in this category.

For illicit opioids, any indication of consumption is considered to be abuse.

For prescription opioids, statements of consumption are not considered to be abuse unless there is some additional evidence suggesting misuse/abuse/nonmedical use. These include:

• Indication that the drug is being consumed for recreational purposes
• Indication that the user was nor prescribed the drug
• Indication for co-ingestion (i.e., the drug is being taken along with something else)
• Use of terms that indicate potential nonmedical use (e.g., popping, snorting)

Examples

• <code>@USERNAME @USERNAME well there's that but i got some heroin, its probably the safest</code>

• <code>Popin percocet; imma nervous wreck!</code>

• <code>I pop percs like i got arthritis</code>

• <code>@USERNAME: @USERNAME i got the sizzurp on deck hahaha Trippin'</code>

• <code>Faded as **** playing Oxy Music in world history class #HowHigh</code>

• <code>I'm on this codeine cause this weed got me coughing</code></td></tr>
<tr><td>Information (I)</td><td>Tweets in which the poster is asking for information or providing information about a drug—prescription or illicit.</td></tr>
</table>

© 2019 Sarker A et al. *JAMA Network Open*

	General statements about the drug may also be put into this class.<br>This category also includes possible medical use and the sharing of news articles that contain information about opioids.<br>Statements about illicit or prescription opioids that do not provide any hint of personal consumption/use are also put in this category.<br><br>**Examples**<br><br>• `An average of 11 people a week die from heroine overdose in Ohio. That is disgusting.`<br>• `I remember when my substitute teacher was on heroin in class... Wait what!`<br>• `2 men were arrested Tues in connection with the selling of heroin out of a State College motel room: [URL] #ImperialMotorInn!`<br>• `I took my codeine and my leg is still killing me and won't let me sleep ????`<br>• `I don't need heroine, nor alcohol, not nicotine, just gib mir benzin!`<br>• `Just found out Kurt cobain was a heroin addict`
**Non-English (N)**	Tweets that are not written in English. If part of the tweet is in English and is relevant to the study (i.e., the opioid-mentioning part is interpretable), then it should be classified into one of the other three categories.<br><br>**Examples**<br><br>• `Te extraño mas mi negra jincha???? te amo`<br>• `El chiva me sabe a té cuando yo toy' loquisimo`
**Unrelated (U)**	Tweets that are not about the drug or opioid, but about something else. Tweets that make metaphorical comparisons (e.g., 'I am addicted to X like heroin') are also included in this category.<br><br>Some examples of the types of tweets in this category include:<br>• handle related (@codeine_XXXX),<br>• heroine (hero),<br>• brown sugar (used for cooking).<br><br>Quoted song lyrics and quotes from movies about opioids should be put in this category as well.<br><br>**Examples**<br><br>• `@USERNAME@USERNAME my favorite infomercial is the oxy clean`<br>• `Yoga and maple brown sugar roasted butternut squash. Tuesdays are alright with me`<br>• `I can't listen to the song Hero/Heroine anymore`<br>• `@codeine_XXXX fool my phone died las night`

© 2019 Sarker A et al. *JAMA Network Open*

	• @eNasty2345 undisputed swag champ that big H be my belt<br>• You are my heroin. -@USERNAME

© 2019 Sarker A et al. *JAMA Network Open*

eTable 2. Optimal Parameter Values for the Different Classifiers Presented

Classifier	Parameters and values	Library used
**Support Vector Machines**	C = 140	Scikit-learn
**Random Forests**	n_estimators = 10,000	Scikit-learn
**k-Nearest Neighbor**	k = 10	Scikit-learn
**Convolutional Neural Network**	Learning rate = 0.001; Dropout = 0.4; batch size = 32	TensorFlow

© 2019 Sarker A et al. *JAMA Network Open*

eTable 3. Class-Specific Recall and Precision, Average Accuracy and Standard Deviation Over Ten Folds for Each Classifier

**Classifier**	**Precision**				**Recall**				**Average accuracy (%)**[a]	**Standard deviation**[b]
	**A**	**I**	**U**	**N**	**A**	**I**	**U**	**N**		
**NB Standard**	0.350	0.481	0.743	0.762	0.543	0.501	0.572	0.837	56.3	2.04
**DT**	0.421	0.522	0.730	0.863	0.414	0.470	0.764	0.881	63.5	1.78
**k-NN**	0.401	0.581	0.694	0.893	0.11	0.081	0.944	0.896	58.8	0.74
**SVMs**	0.526	0.678	0.722	0.938	0.422	0.489	0.860	0.905	69.3	1.25
**RF**	0.524	0.672	0.728	0.936	0.434	0.496	0.855	0.911	69.5	1.59
**CNN**[c]	0.515	0.661	0.736	0.928	0.451	0.506	0.842	0.920	69.4	1.52

[a] Averaged over 10 folds.

[b] Of accuracy over 10 folds.

[c] Although parameter optimization was performed for the CNN using separate train and validation sets, for these results, the optimal parameters were used in a 10-fold cross validation experiment.

© 2019 Sarker A et al. *JAMA Network Open*

eTable 4. Opioid Keywords and Spelling Variants

oxycontin oxicontin oxcotin oycotin oxycotins oycontin oxycontins oxycoton oxicotin ocycotin oxycodin oxycottin oxycotine ocycontin oxycintin oycotine oxcycontin roxicontin oxycotin oxycontine roxycontin oxycotton oxycottin oxycoton

percocet percocet10 perocets percosets percacet percocetes percet pecocet percacets perocet percoct percocets pecocets percoet percocit percocoet percot percoset percecet percicet

dilaudid dialudid dilaudad diluadid diaudid dilaudin dilauded dilauid dillaudid dilaudids dulaudid diludid diladid dialaudid dilaudud dalaudid dilautid dilaud dilladid

tramadol trammadol tramadal tramal tramdol tramadols tramado tramedol tramadoll tramadole tramidol tamadol tranadol tramodol tremadol tramamdol

demmies

ultram ultam ultramer

percodan

oxycodone oxycodons oycondone oycodone oxycodine roxicodone oxicodone oxycoton ocycodone oxcycodone oxycodne oxycodones oxycodin oyxcodone roxycodone oxcodone oxycondone oxycoden oxycodon oxyxodone oxycodene

hydromorphone hydromorphine

duramorph

fentanyl fentanly fentayl fentynyl fentonyl fentanayl fentanal fentnayl fentany fentenyl fental fentyl fenanyl fentinyl fentnyl fentanl fetanyl fentnyal fentanyal fentanol

meperidine

vicodin vicoden vycodin

fizzies

narcan

oxymorphone

hydros

amidone

morphine morphin morpheine morhphine moraphine diamorphine mophine morephine morhine morphone morhpine morphiene moriphine morpine morphene morphones morphein morophine morphines

demerol demarol demorol depomedrol demeral

dolophine

codeine codiene codeine coedine codine codene codein

heroin herroine heroins heroine heorin herion

methadone methadones methadose methodone mehtadone metadone methadon

357s

512s o bomb

big h

black tar

brown sugar

captain cody

china girl

china white

© 2019 Sarker A et al. *JAMA Network Open*

chiva<br>doors & fours<br>hell dust<br>mexican brown<br>miss emma<br>murder 8<br>negra<br>norco<br>opana<br>oxy<br>oxy 80s<br>oxycet<br>oxys<br>percs<br>purple drank<br>sizzurp<br>skag<br>tango and cash<br>vicos<br>vics<br>vike<br>watson-387<br>white horse

© 2019 Sarker A et al. *JAMA Network Open*

eTable 5. Distribution of Tweet Classes Across the Training and the Evaluation Sets

Note: The sum of the %s for the totals for illicit and prescription opioids is above 100. This is because the same tweet may contain both illicit and prescription opioid mentions.

	**Abuse (A)**	**Information (I)**	**Unrelated (U)**	**Non-English (N)**	**Total**
**Training**	1430 (19.85%)	1585 (22.0%)	3852 (53.47%)	337 (4.68%)	7204 (80%)
**Test**	318 (17.65%)	416 (23.09%)	978 (54.27%)	90 (4.99%)	1802 (20%)
**Illicit opioids**	1270 (18.04%)	1469 (20.87%)	3881 (55.14%)	418 (5.94%)	7038 (78.1%)
**Prescription opioids**	557 (24.68%)	685 (30.35%)	1005 (44.53%)	10 (0.44%)	2257 (25.1%)

© 2019 Sarker A et al. *JAMA Network Open*

eTable 6. Counties Within Each Substate in Pennsylvania

Substate region	Number of counties in substate region	Counties
R 1	1	Allegheny
R 3,8,9,51	6	Beaver Butler Cambria Armstrong Clarion Indiana
R 4, 11, 37, 49	6	Berks, Carbon, Monroe, Pike, Schuylkill, Wayne
R 5, 18, 23, 24, 46	9	Blair, Cumberland, Perry, Franklin, Fulton, Huntingdon, Juniata, Mifflin, Bedford
R 6, 12, 16, 31, 35, 45, 47	12	Bradford, Sullivan, Centre, Columbia, Montour, Snyder, Union, Clinton, Lycoming, Northumberland, Tioga, Potter
R 7, 13, 20, 33	4	Bucks, Chester, Delaware, Montgomery
R 10, 15, 27, 32, 43, 44	10	Cameron, Elk, McKean, Clearfield, Jefferson, Lawrence, Mercer, Forest, Warren, Venango
R 17, 21	2	Erie, Crawford
R 19, 26, 28, 42	5	Dauphin, Lancaster, Lebanon, Adams, York
R 22, 38, 40, 41, 48	5	Fayette, Somerset, Washington, Westmoreland, Greene
R 29, 39	2	Lehigh, Northampton
R 30, 50	4	Luzerne, Wyoming, Lackawanna, Susquehanna
R 36	1	Philadelphia

© 2019 Sarker A et al. *JAMA Network Open*

eTable 7. Confusion Matrices Illustrating Common Errors Made by the 2 Best Performing Systems (Ensemble_1 and Ensemble_biased_1 in Table 1)

	**Ensemble_1**					**Ensemble_biased_1**			
	**A**	**U**	**I**	**N**		**A**	**U**	**I**	**N**
**A**	135	145	36	2		161	123	32	2
**U**	67	847	55	9		90	818	61	9
**I**	59	122	235	0		78	104	234	0
**N**	0	4	0	86		0	4	0	86

© 2019 Sarker A et al. *JAMA Network Open*
